# Contributions of the Hippocampal CA3 Circuitry to Acute Seizures and Hyperexcitability Responses in Mouse Models of Brain Ischemia

**DOI:** 10.3389/fncel.2018.00278

**Published:** 2018-08-29

**Authors:** Hongmei Song, Sivakami M. Mylvaganam, Justin Wang, Saeyon M. K. Mylvaganam, Chiping Wu, Peter L. Carlen, James H. Eubanks, Jiachun Feng, Liang Zhang

**Affiliations:** ^1^Krembil Research Institute, University Health Network, Toronto, ON, Canada; ^2^Department of Neurosurgery, The First Hospital of Jilin University, Changchun, China; ^3^Department of Medicine (Neurology), University of Toronto, Toronto, ON, Canada; ^4^Department of Physiology, University of Toronto, Toronto, ON, Canada; ^5^Department of Surgery (Neurosurgery), University of Toronto, Toronto, ON, Canada; ^6^Department of Neurology, The First Hospital of Jilin University, Changchun, China

**Keywords:** brain slices, CA3, discharges, EEG, epileptiform, EPSC, IPSC, ischemia

## Abstract

The hippocampal circuitry is widely recognized as susceptible to ischemic injury and seizure generation. However, hippocampal contribution to acute non-convulsive seizures (NCS) in models involving middle cerebral artery occlusion (MCAO) remains to be determined. To address this, we occluded the middle cerebral artery in adult C57 black mice and monitored electroencephalographic (EEG) discharges from hippocampal and neocortical areas. Electrographic discharges in the absence of convulsive motor behaviors were observed within 90 min following occlusion of the middle cerebral artery. Hippocampal discharges were more robust than corresponding cortical discharges in all seizure events examined, and hippocampal discharges alone or with minimal cortical involvement were also observed in some seizure events. Seizure development was associated with ipsilateral hippocampal injuries as determined by subsequent histological examinations. We also introduced hypoxia-hypoglycemia episodes in mouse brain slices and examined regional hyperexcitable responses *ex vivo*. Extracellular recordings showed that the hippocampal CA3 region had a greater propensity for exhibiting single/multiunit activities or epileptiform field potentials following hypoxic-hypoglycemic (HH) episodes compared to the CA1, dentate gyrus, entorhinal cortical (EC) or neocortical regions. Whole-cell recordings revealed that CA3 pyramidal neurons exhibited excessive excitatory postsynaptic currents, attenuated inhibitory postsynaptic currents and intermittent or repetitive spikes in response to HH challenge. Together, these observations suggest that hippocampal discharges, possibly as a result of CA3 circuitry hyperexcitability, are a major component of acute NCS in a mouse model of MCAO.

## Introduction

Stroke (either hemorrhagic or ischemic) is a common cause of seizures/epilepsy in adult and aging populations (Jetter and Cavazos, 2008; Bladin and Bornstein, 2009; Brodie et al., 2009; Menon and Shorvon, 2009; Chen et al., 2010; Guekht and Bornstein, 2012; Procaccianti et al., 2012; Chung, 2014). Early-onset seizures are primarily observed within the first 24 h following a stroke and are considered a medical emergency as life-threatening status epilepticus may follow (Waterhouse et al., 1998). These seizures can be expressed as generalized convulsive seizures (CS) or focal/generalized non-convulsive seizures (NCS) that require electroencephalography (EEG) for diagnosis (Silverman et al., 2002; Jordan, 2004; Claassen et al., 2007; Mecarelli et al., 2011; Mikell et al., 2016). The development of seizures following stroke is associated with a worse prognosis including higher rates of extended hospitalization, long-term disability and death (Szaflarski et al., 2008; Burneo et al., 2010; Mecarelli et al., 2011; Gilad, 2012; Jauch et al., 2013; Huang et al., 2014; Bryndziar et al., 2016; Xu et al., 2016). Despite this link between seizure development and poorer patient outcomes, specific treatment guidelines for post-stroke seizures have yet to be established. They may depend on a better understanding of seizure pathogenesis. Stroke severity and cortical involvement are currently recognized risk factors for seizure genesis following ischemic stroke (Jetter and Cavazos, 2008; Bladin and Bornstein, 2009; Brodie et al., 2009; Menon and Shorvon, 2009; Chen et al., 2010; Guekht and Bornstein, 2012; Procaccianti et al., 2012; Chung, 2014). However, while seizures after ischemic stroke are thought to be originated from penumbra areas, the underlying pathophysiological process is not well understood (Pitkänen et al., 2016; Kelly, 2017; Reddy et al., 2017; Sommer, 2017).

Animal models have contributed greatly to our understanding of post-stroke seizures and our continued exploration of potential treatment strategies that may be eventually translated to humans (Pitkänen et al., 2016; Kelly, 2017; Reddy et al., 2017; Sommer, 2017). Previous studies have examined acute seizures in rat models of unilateral middle cerebral artery occlusion (MCAO; Hartings et al., 2003; Williams et al., 2004, 2006; Karhunen et al., 2006; Lu et al., 2009, 2013; Cuomo et al., 2013; Kelly, 2017), photothrombotic ischemia (Karhunen et al., 2007; Paz et al., 2013; Lippmann et al., 2017) and intra-hippocampal endothelin-1 infusion (Tsenov et al., 2015). Other studies have examined late-onset epileptic seizures in rat models of brain ischemia (Kadam et al., 2010; Kelly, 2017). Mice have been increasingly used in stroke research largely because genetic and molecular manipulations in mouse models offer great advantages in mechanistic investigation. While mouse models produce similar brain injuries as compared with rat models (Fluri et al., 2015), studies of post-ischemic seizures in adult mice remain relatively limited (Barth and Mody, 2011; El-Hayek et al., 2011; Wang et al., 2015; Wu et al., 2015).

Several studies have detailed acute NCS or EEG discharges in the absence of convulsive motor behaviors in adult rats following MCAO. These studies indicated that NCS originated predominantly from the hemisphere ipsilateral to MCAO and that NCS are an aggravating factor of ischemic brain injury (Hartings et al., 2003; Williams et al., 2004, 2006; Karhunen et al., 2006; Lu et al., 2009, 2013; Cuomo et al., 2013). A recent study of our lab examined acute seizures in a mouse model of MCAO (Wu et al., 2015). While focusing on CS, this study also revealed NCS that featured hippocampal and cortical EEG discharges. Our present experiments further examine such NCS. Specifically, in view of hippocampal seizure activities observed in other models of brain ischemia (Caruana et al., 2008; Barth and Mody, 2011; Tsenov et al., 2015; Lippmann et al., 2017), we hypothesized hippocampal discharges are a major component of NCS in adult mice following MCAO. As the CA3 circuitry is known to play a critical role in generation of hippocampal epileptiform activity (Beenhakker and Huguenard, 2009; Le Duigou et al., 2014), we also hypothesized that relative to other hippocampal or cortical areas, the CA3 region is susceptible to hyperexcitable responses when exposed to hypoxic-hypoglycemic (HH) episodes. Overall, data we collected from *in vivo* and *ex vivo* experiments are supportive of these hypotheses.

## Materials and Methods

### Animals

Male C57 black mice (C57BL/6J) were obtained from Charles River Laboratory (Senneville St-Constant, QC, Canada) and housed in a local vivarium that was maintained between 22°C and 23°C and with a 12-h light on/off cycle. We used adult mice aged 6–12 or 5–9 months for *in vivo* or *ex vivo* experiments, as their ages may roughly correspond to age 30–40 years in humans (Flurkey et al., 2007). We also used young mice ages 1–2 months for *ex vivo* experiments because brain tissues of young animals are generally better preserved *ex vivo*. This provides greater probability of examining population epileptiform activities compared to brain tissues of adult animals (Bernard, 2006). All of the experimentations conducted in this study were reviewed and approved by the animal care committee of the University Health Network as per the guidelines of the Canadian Council for Animal Care.

### EEG Recordings and Data Analysis

Electrode construction and implantation were performed as previously described (El-Hayek et al., 2011; Wang et al., 2015; Wu et al., 2015). All electrodes were made with polyimide-insulated stainless-steel wires (outside diameter 0.2 mm, Plastics One, Roanoke, VA, USA). Recording electrodes were implanted bilaterally or unilaterally in hippocampal and parietal cortical areas. The stereotaxic coordinates were 2.3 mm posterior, 2.0 mm lateral and 2.0 mm ventral to Bregma for hippocampal electrodes and 0.6 mm posterior, 1.5 mm lateral and 1.0 mm ventral to Bregma for cortical electrodes (Franklin and Paxinos, 1997). A reference electrode was positioned into a frontal area (2.0 mm anterior, 1.0 mm lateral and 0.5 mm ventral to Bregma). Implanted mice were allowed to recover for ≥1 week prior to baseline monitoring. The locations of implanted electrodes were verified by subsequent histological assessments if suitable (Supplementary Figure S1F).

All recordings were made from freely moving mice. EEG signals were collected using a dual-channel AC microelectrode amplifier with input frequencies set in a range of 0.1–1,000 Hz and amplification at 1,000 × (AM Systems, model 1800). Data digitization (5 or 10 KHz), acquisition, storage and analysis were done using Digidata 1300 or 1400 and pClamp software (Molecular Devices; Sunnyvale, CA, USA).

EEG root mean square (RMS) was used to quantify signal powers as this parameter has been shown to be a sensitive measure of EEG changes in mouse models of bran ischemia (El-Hayek et al., 2011; Wang et al., 2015; Wu et al., 2015) and a rat model of brain hemorrhage (Klahr et al., 2015). RMS values were calculated from continuous 30–90 s data segments as previously described (El-Hayek et al., 2011). Briefly, power spectra were generated (rectangle function) by averaging with 50% window overlap and a spectral resolution of 0.3 Hz. RMS was calculated in pClamp by taking the square root of the integral of the power spectrum. Concurrent hippocampal and cortical signals of same durations were used to calculate EEG signal powers. Baseline (before MCAO) RMS values were calculated from 30-s data segments collected during stable behavioral immobility. Pre-discharge RMS values were calculated from 30-s segments collected immediately before the onset of discharges. Discharge RMS values were calculated from subsequent data segments. In the case of NCS with minimal cortical involvement, cortical data segments corresponding to concurrent hippocampal discharges were used for RMS calculation.

### Middle Cerebral Artery Occlusion (MCAO)

Permanent and reversible MCAO (pMCAO and rMCAO) were performed using intra-luminal suture methods as previously described (Wu et al., 2015). To induce pMCAO, a silicon-coated fine suture (#6, Doccol Corporation, Redlands, CA, USA) was inserted into the common carotid artery and advanced through the internal carotid artery with its tip 7–8 mm distal to the carotid bifurcation. The common carotid artery together with the inserted suture was then irreversibly ligated. To induce rMCAO, the suture was inserted through the external carotid artery and advanced through the internal carotid artery. After suture placement, the external carotid artery was temporally ligated and skin wound was loosely sutured. The animal was allowed to recover from anesthesia but with the inserted suture for 45, 60 or 90 min, referred to as rMAO_45 min_, rMCAO_60 min_ or rMCAO_90 min_, respectively. To induce permanent MCAO via electrocoagulation (pMCAOe), a skin incision was made to expose the skull in the area between the right ear and eye. A small hole was drilled through the skull, and the main ascending branch of the middle cerebral artery was coagulated using a clinically utilized high temperature cautery (Bovie, Clearwater, FL, USA). Age-matched mice that experienced isolation of the middle cerebral artery but no artery occlusion were used as sham controls.

### Seizure Detection

Baseline monitoring was performed ≥1 week after electrode implantation, and individual mice were under continuous visual inspection and EEG monitoring for up to 6 h during this monitoring. To detect acute seizures following MCAO, mice were under continuous behavioral inspection and EEG recordings for 4–6 h post-surgery as well as overnight video monitoring for another 10–14 h. Behavioral inspection and EEG monitoring were resumed next day and at later time points if possible.

CS were recognized by vigorous motor behaviors including fast running, jumping, barrel rolling and/or falling (loss of righting reflex) with frequent limb spasms (El-Hayek et al., 2011; Wang et al., 2015; Wu et al., 2015). NCS were defined by ictal-like EEG discharges that occurred in a state of behavioral arrest. These discharges displayed repetitive singe-spike and poly-spike waveforms with durations of ≥5 s and amplitudes at least two times greater than background signals (Wais et al., 2009; Wu et al., 2015; Song et al., 2018). EEG spikes were recognized by clusters of single spikes that occurred during behavioral immobility and displayed durations ≥3 s and amplitudes that were at least two times of greater than background signals.

### Brain Histology

Mice were euthanized within 24 h following MCAO or 4–5 weeks post sham surgery for preparation of brain histological sections (El-Hayek et al., 2011; Wang et al., 2015; Wu et al., 2015). Briefly, mice were anesthetized via an intra-peritoneal injection of sodium pentobarbital (100 mg/kg) and trans cardially infused with 10% neutral buffered formalin solution. Brains were removed and further fixed in a hypertonic (with 20% sucrose) formalin solution. Cryostat coronal sections of 30 μm thickness were obtained and stained with cresyl violet. Images of brain sections were obtained using a slide scanner (Aperio digital pathology slide scanner AT2, Leica) at 20× magnification and analyzed using ImageScope (Leica) and ImageJ (National Institute of Health, Bethesda, MD, USA) software.

### Brain Slice Preparation and *ex vivo* Perfusion

Brain slice preparation and electrophysiological recordings *ex vivo* were conducted as previously described (Wais et al., 2009; El-Hayek et al., 2011; Moradi-Chameh et al., 2014). Briefly, mice were anesthetized using sodium pentobarbital (70 mg/kg, intraperitoneal injection) and trans cardially infused with cold, dissection-only artificial cerebrospinal fluid (ACSF) before brain dissection. Brains were quickly removed and sliced (0.4 mm thickness) using a vibratome (Leica VT1200) in ice-cold dissection-only ACSF. Horizontal slices from the ventral part of brain were used to examine hippocampal and entorhinal activities, and coronal slices from the rostral part of the brain were used to examine neocortical activities. After slicing, slices were maintained in oxygenated (5%CO_2_-95%O_2_) standard ACSF for 1–6 h before recordings. The components of dissection-only ACSF were (in mM): sucrose 280, KCl 3.5, CaCl_2_ 0.5, MgCl_2_ 6, HEPES 10 and D-glucose 10. The components of standard ACSF were (mM): NaCl 125, KCl 3.5, NaH_2_PO_4_ 1.25, CaCl_2_ 2, MgSO_4_ 1.3, NaHCO_3_ 25 and D-glucose 10. The components of modified ACSF for inducing HH episodes were (mM); NaCl 125, KCl 4.5, NaH_2_PO_4_ 1.25, CaCl_2_ 2, MgSO_4_ 1.3, NaHCO_3_ 25 and D-glucose 1. If needed, kynurenic acid (Sigma-Aldrich, Oakville, ON, Canada), a general blocker of ionotropic glutamate receptors, was added into standard and modified ACSFs at 3 mM to suppress the activity of ionotropic glutamate receptors.

All recordings were done in a submerged chamber and at a perfusate temperature of 36°C. ACSF was applied at a high rate (~15 ml/min), and both the top and bottom surfaces of the slice were exposed to the perfusate. Humidified gas of 95%O_2_-5%CO_2_ was allowed to pass over the perfusate to increase local oxygen tension. Previous studies have shown that a fast, top and bottom perfusion of the slice is important for maintaining spontaneous population activities under submerged conditions (Wu et al., 2005a,b; Hájos and Mody, 2009). Slices were perfused with modified ACSF during HH episodes. A mixed gas of 10%O_2_-5%CO_2_-85%N_2_ was used to aerate modified ACSF and to apply over the perfusate. HH episodes in individual slices were 12–13 min.

### Recordings and Afferent Stimulation in Slices

Recording electrodes were made with thin-wall glass tubes (World Precision Instruments, Sarasota, FL, USA). Extracellular electrodes were filled with a solution containing 200 mM NaCl and 2 mM HEPES (pH 7.4; resistance of 1–2 MΩ). Patch electrodes were filled with a solution containing 140 mM potassium gluconate, 10 mM KCl, 2 mM HEPES and 0.1 mM EGTA (pH 7.25 and resistance of 4–5 MΩ). A modified patch pipette solution with “high [Cl^−^]” was used in some experiments, by decreasing potassium gluconate to 50 mM and increasing KCl to 100 mM while keeping other components unchanged. All chemicals for making patch pipette solution were obtained from Sigma-Aldrich (Oakville, ON, Canada). Extracellular and intracellular signals were collected using a dual channel amplifier (Multiclamp 700A or 700BA, Molecular Devices). Data digitization, acquisition, storage and analyses were done using Digidata 1,400 and pClamp software.

Bipolar electrodes made of polyimide-insulated stainless steel wires (outer diameter 0.1 mm) were used for afferent stimulation. Constant current pulses (0.1 ms duration, at a near maximal intensity of 150 μA) were generated by a Grass stimulator and delivered through a photoelectric isolation unit (S88, Natus Neurology Incorporated—Grass Products, Warwick, RI, USA). An electrode was placed in the CA3 stratum radiatum or oriens area to elicit CA3 and CA1 responses or in the middle layer of entorhinal or neocortical area to evoke local responses. Twin stimuli with identical intensities and an interval of 30 ms or 4 ms were used to assess paired enhancement or suppression. Afferent stimulations were applied every 20 s for 2–3 min before HH and after termination of HH or continuously before and following HH.

### *Ex vivo* Data Analysis

Slices that displayed stably evoked field potentials (≥0.2 or 0.5 mV for cortical or hippocampal responses) during baseline monitoring were used for subsequent examinations of HH responses. Single/multiunit activities were defined as extracellularly recorded spikes with amplitudes ≥4 times of the standard deviation of baseline signals and spike durations of ≤5 ms. Repetitive field potentials were recognized as intermittent events with complex waveforms, durations of 20–200 ms and amplitudes ≥2 times greater than background signals. An event detection function (threshold search method) of pClamp was used to automatically detect unit activities or repetitive field potentials. Original data were treated with a band-pass filter before event detection (2–1,000 Hz or 0.2–200 Hz for unit activity or repetitive field potentials, respectively). Detected events were visually inspected and artifacts were excluded (Wu et al., 2005a,b; Wais et al., 2009; El-Hayek et al., 2011). Spreading depression (SD) like responses (Somjen, 2001) were defined as downward (negative) shifts in extracellular potentials with magnitudes of ≥2 mV and a base-to-peak time of ≤15 s. To compare evoked field potentials collected before HH and after termination of HH, measurements were made from averages of 4–5 consecutive responses. To assess changes in evoked field potentials during HH, measurements were made from individual responses and data were presented as percentiles of baseline mean amplitudes. The amplitude ratio (%) of the 2nd vs. 1st evoked field potentials was used to determine paired enhancement or suppression.

Repetitive EPSCs and IPSCs with simple and/or overlapped waveforms, amplitudes of ≥15 pA and intervals of >10 ms were automatically detected using Mini Analysis Program (Synaptosoft, Decatur, GA, USA). Detected events were visually verified and the false events were rejected. Under the experimental conditions, these synaptic currents displayed complex or overlapping waveforms particularly following HH, disallowing assessments of their rising and decay times. We thus analyzed inter-event intervals and areas (pA/ms) of EPSC and IPSC events as a general estimation of total synaptic activity in individual neurons before and following HH.

Resting membrane potentials, input resistance and action potentials of CA3 pyramidal neurons were measured as per previous studies of our lab (Wu et al., 2005a; Moradi-Chameh et al., 2014). A voltage ramp protocol (Chung et al., 1998) was used to assess chord conductance and reversal potentials of changed holding currents. In these experiments, CA3 pyramidal neurons were stimulated with repetitive voltage ramps every 20 s (from a holding potential of −60 mV to −100 mV and then to −40 mV, at a speed of 20 mV/s). Holding currents were measured from 1-s segments collected before each voltage ramp. Chord conductance was estimated over the ramp voltage range of −60 mV to −100 mV. The reversal potential of the HH-induced outward holding current was determined according the voltage at which baseline and post-HH ramp currents crossed over in the voltage range of −60 mV to −100 mV. Three consecutive ramp currents, collected before and following HH for 5–7 or 6–9 min, were averaged for the above measures. We did not estimate the reversal potential of HH-induced inward current in the present experiments due to complications of spike currents induced by positive ramp voltages.

### Statistical Analysis

A Student’s *t*-test or Mann-Whitney rank sum test was used for two group comparisons (SigmaStat software, Systat Software Inc, San Jose, CA, USA). For multiple group comparisons, a one-way ANOVA was employed, followed by a multiple comparison test (control vs. test or pairwise). For proportional comparisons, a Chi-square or Fischer exact test was used. Data were presented as mean and standard error of mean (SEM) throughout the text and figures.

## Results

### NCS Observed From Adult Mice Following MCAO

NCS and CS were observed in 49 of 67 mice following MCAO (Table 1), but neither seizure nor aberrant EEG activity was found in 10 control mice following sham surgery. NCS were recognized by ictal-like hippocampal-cortical EEG discharges that occurred while mice were in a state of behavioral arrest or immobility. These discharges displayed repetitive spike waveforms with amplitudes of 0.5–6 mV and durations of 10–70 s and encompassed strong rhythmic signals between 5 Hz and 300 Hz. EEG spikes were observed from hippocampal recordings in seven mice during behavioral immobility, with lower amplitudes (≤0.5 mV) and briefer durations (8.9 ± 2.9 s nine events) relative to the ictal-like discharges. Contrary to NCS, CS manifested with vigorous motor behaviors including fast running, jumping, barrel rolling and/or falling (loss of righting reflex). CS occurrence was not associated with evident discharges in hippocampal-cortical recordings, suggesting that deeper subcortical structures might be involved in CS genesis (Wang et al., 2015; Wu et al., 2015). We focused on NCS in the present experiments as CS have already been detailed (Wu et al., 2015).

**Table 1:** Non-convulsive seizures (NCS) and convulsive seizures (CS) observed following MCAO.

	Mice with or without acute seizures				
	NCS + CS	CS only	NCS only	No seizure	Numbers of mice examined per protocol
rMCAO_45 min_	5	6		6	17
rMCAO_60 min_	6	4		3	13
rMCAO_90 min_	7	7		4	18
pMCAO	7	7		5	19
pMCAOe			2	6	8
Sham surgery				10	10

*rMCAO_45 min_, rMCAO _60 min_ and rMCAO_90 min_—reversible middle cerebral artery occlusion via intraluminal suture insertion for 45, 60 or 90 min, respectively. pMCAO, permanent middle cerebral artery occlusion via intraluminal suture insertion. pMCAOe, permanent middle cerebral artery occlusion via electrocoagulation*.

#### Incidences and Latencies of NCS and Their Relation to CS

**Figure 1:**
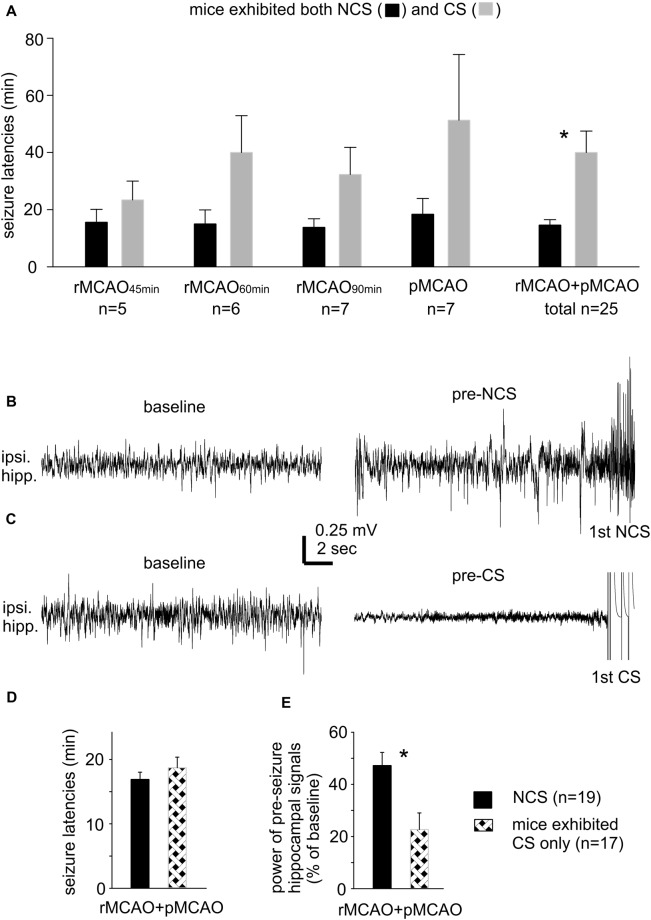
Seizure latencies and pre-seizures electroencephalography (EEG) signals. **(A)** Non-convulsive seizures (NCS) and convulsive seizures (CS) latencies measured from mice that exhibited both types of seizures following different middle cerebral artery occlusion (MCAO) protocols via intraluminal suture insertion. *, NCS vs. CS. **(B,C)** Representative traces collected from two mice; ipsilateral hippocampal signals sampled during baseline monitoring (before MCAO) and immediately before the onset of the first NCS or CS (pre-seizure). **(D,E)** Data collected from two groups of mice with NCS or CS as the first detected seizure within 30 min following MCAO. Seizure latencies were determined from termination of surgery to the onset of the first seizure. Pre-seizure signal powers were normalized as percentiles of baseline measures. *, pre-NCS vs. pre-CS signals. Here and in following figures, ipsilateral and contralateral are in reference to unilateral MCAO; data are presented as mean ± SEM except where specified; *indicates significance at *p* < 0.05.

There was no significant difference in the incidence of NCS and CS in mice that experienced different MCAO protocols through intraluminal suture insertion. Of 11–15 mice examined following rMCAO_45 min_, rMCAO_60 min_, rMCAO_90 min_ or pMCAO, NCS and CS were both observed in five to seven mice and CS was observed alone in the other 4–8 mice (Table 1). NCS, but not CS, were also detected in 2/8 mice following permanent MCAO via electrocoagulation (pMCAOe). Of the acute seizures observed following rMCAO protocols, all NCS occurred prior to suture withdrawal whereas CS arose either before and/or after suture withdrawal. In addition, the occurrence of NCS always preceded that of CS and never followed CS in mice that exhibited both types of seizures. Seizure latencies, determined from termination of surgery to the onset of the first seizure, were 14.6 ± 1.9 min and 40.0 ± 7.5 min for NCS and CS in these mice (*n* = 25; *p* < 0.001; Figure 1A). Together, these observations suggest that NCS are an early (relative to CS) seizure marker or a predictor of subsequent CS in our model. However, other factors may underlie CS genesis without preceding NCS in some mice following MCAO.

Our lab previously examined acute CS in adult and aging mice following ischemia-hypoxia episodes (El-Hayek et al., 2011; Wang et al., 2015). CS genesis in this model was not preceded by NCS but associated with severe suppression of ipsilateral hippocampal EEG signals. Since CS observed following ischemia-hypoxia or MCAO are similar in behavioral manifestations (Wang et al., 2015; Wu et al., 2015), we questioned whether mice with CS alone following MCAO may also experience severe EEG suppression. We thus compared ipsilateral hippocampal EEG signals between mice with NCS or CS as the first detected seizure post pMCAO/rMCAO (*n* = 19 or 17 mice). Examples of ipsilateral hippocampal traces from two mice are presented in Figures 1B,C showing similar baseline signals (before MCAO; left) but remarkable suppression of pre-CS relative to pre-NCS EEG signals (right). Values of RMS of ipsilateral hippocampal signals were calculated from EEG segments (30-s) collected during baseline monitoring and immediately before the first NCS or CS, and data were presented as percentiles of baseline RMS. Seizure latencies were similar in these two groups (16.9 ± 1.1 min for NCS and 18.7 ± 1.6 min for CS, *n* = 19 and 17 mice, *p* = 0.360; Figure 1D), but suppression of ipsilateral hippocampal EEG was more severe for the pre-CS relative to the pre-NCS signals (22.7 ± 6.3% vs. 47.2 ± 5.0% of baseline, *p* < 0.001; Figure 1E). These observations are in keeping with our previous studies of acute CS in mice following hypoxia-ischemia episodes (El-Hayek et al., 2011; Wang et al., 2015). We therefore speculate that severe suppression of brain activity soon after MCAO challenge may partly explain why some mice exhibited CS without preceding CS.

#### NCS Featured With Robust Hippocampal Discharges

We used different EEG recording protocols to compare hippocampal and cortical contributions to NCS. Specifically, individual mice were monitored unilaterally from ipsilateral hippocampal and parietal cortical areas or bilaterally from hippocampal and parietal cortical areas. In general, hippocampal discharges displayed low but gradually increased signals upon onset, followed by repetitive single and poly-spike waveforms with large amplitudes and finally abrupt termination with a phase of post-discharge signal suppression lasting up to several seconds. Corresponding cortical discharges showed variable waveforms. To compare hippocampal and cortical signal power, RMS values were calculated from segments collected before MCAO, immediately before the onset of discharges and during discharges (30–85 s in length).

**Figure 2:**
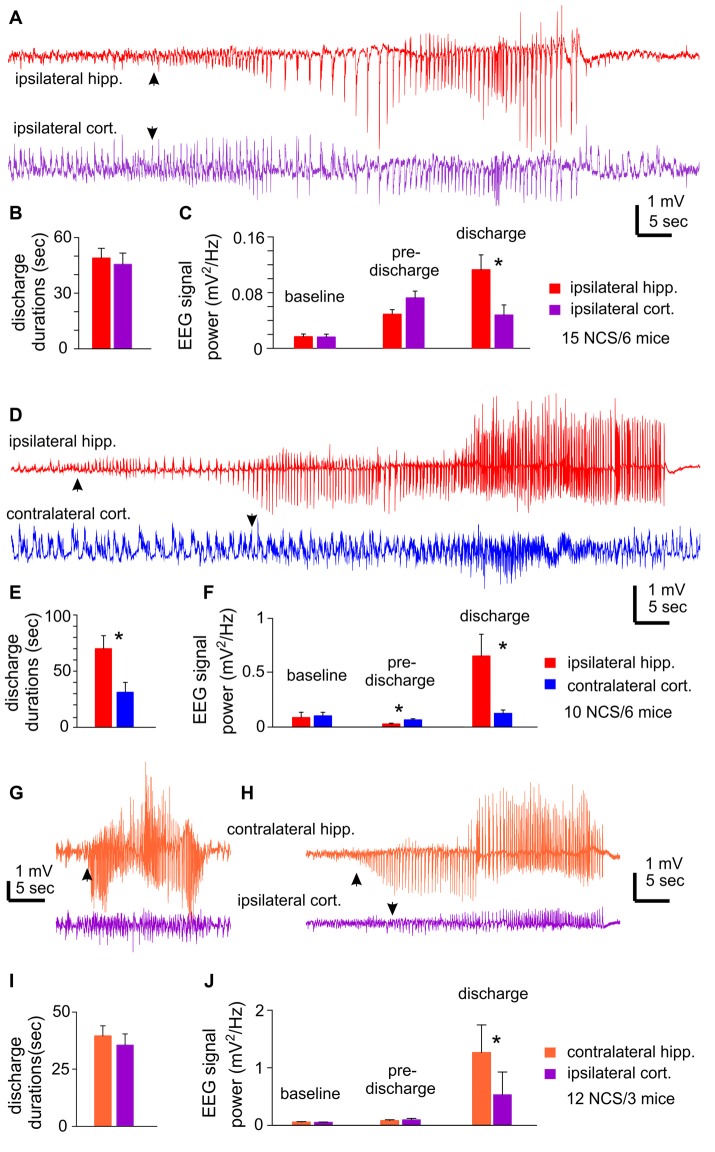
NCS observed following MCAO via intraluminal suture insertion. **(A–C)** Data collected via unilateral EEG recordings from ipsilateral hippocampal and parietal cortical areas. Representative discharges sampled from a mouse following reversible MCAO for 60 min. **(D–F)** Data collected via bilateral recordings from ipsilateral hippocampal and contralateral cortical areas. Representative discharges sampled from a mouse following permanent MCAO. **(G–J)** Data collected via bilateral recordings from contralateral hippocampal and ipsilateral cortical areas. Representative discharges sampled from two mice following permanent MCAO **(G)** or reversible MCAO for 90 min **(H)**. In all illustrated traces, discharge onsets are denoted by filled arrows, and hippocampal discharge termination recognized by suppressed or reduced signals. Baseline signal powers were calculated from 30-s data segments collected during stable behavioral immobility. Pre-discharge signal powers were calculated from 30-s segments collected immediately before the onset of discharges. Discharge signal powers calculated from corresponding data segments. In case of NCS with minimal cortical involvement, cortical signal powers were calculated from data segments that corresponded to concurrently recorded hippocampal discharges. *, *p* < 0.05, ipsilateral vs. contralateral.

Unilateral EEG recordings from ipsilateral hippocampal and cortical areas detected 15 NCS events from six mice. One example of detected NCS is presented in Figure 2A, where the illustrated hippocampal discharge displayed a “typical” pattern as described above but the onset and termination of corresponding cortical discharge were not clearly definable. Measured durations of ipsilateral hippocampal and cortical discharges were not significantly different (49.0 ± 5.2 vs. 45.6 ± 6.0 s *p* = 0.675; Figure 2B), but some cortical discharges might be over- or under-estimated. RMS at baseline and preceding discharge were not significantly different between hippocampal and cortical measures, but RMS of hippocampal discharges was greater than that of cortical discharges (0.113 ± 0.021 vs. 0.048 ± 0.014 mV^2^/Hz; *p* = 0.009; Figure 2C).

Bilateral EEG recordings from ipsilateral hippocampal and contralateral cortical areas detected 10 NCS events in six mice. Of these NCS, seven events showed concurrent hippocampal and cortical discharges while the other three events from two mice presented hippocampal discharges alone or with minimal cortical involvement (Figure 2D). The ipsilateral hippocampal discharges were longer in duration and greater in RMS relative to the contralateral cortical discharges (70.0 ± 11.5 vs. 31.2 ± 8.8 s and 0.654 ± 0.20 vs. 0.126 ± 0.031 mV^2^/Hz, *n* = 10; *p* = 0.016 and 0.017); whereas RMS before discharge was greater for the contralateral cortical signals relative to the ipsilateral hippocampal signals (Figures 2E,F).

Bilateral EEG recordings from contralateral hippocampal and ipsilateral cortical areas detected 12 NCS from three mice. Two of the 12 events showed weaker or minimal cortical discharges as compared to hippocampal discharges (Figures 2G,H). Overall the hippocampus and cortical discharges were similar in durations (39.6 ± 4.4 vs. 35.6 ± 4.9 s *p* = 0.548; Figure 2I), but corresponding RMS were greater for the contralateral hippocampal discharges relative to the ipsilateral cortical discharges (1.265 ± 0.479 vs. 0.532 ± 0.394 mV^2^/Hz; *p* ≤ 0.001; Figure 2J).

**Figure 3:**
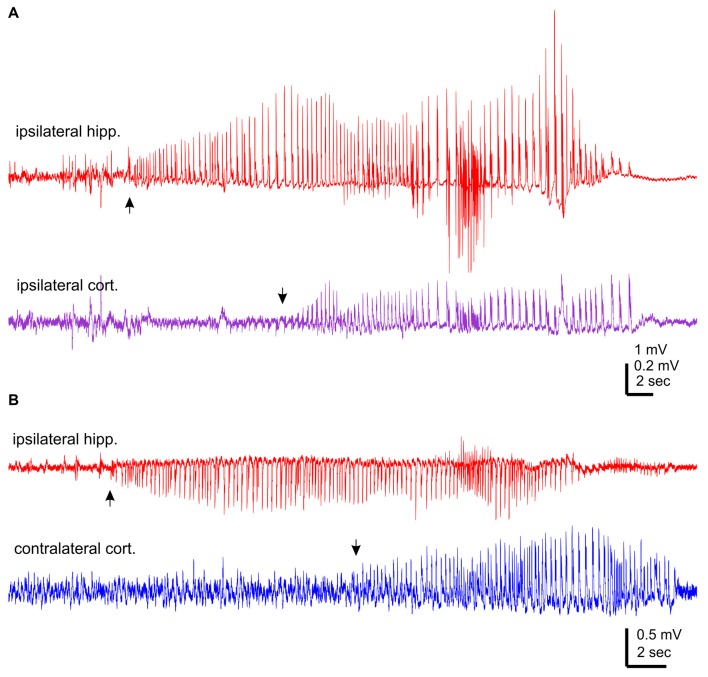
NCS observed following permanent MCAO via electrocoagulation. Traces were collected from two mice. Hippocampal-cortical discharges recorded unilaterally from ipsilateral hippocampal and cortical areas **(A)** or bilaterally from ipsilateral hippocampal and contralateral cortical areas **(B)**. Discharge onsets denoted by filled arrows.

The aforementioned NCS were observed from mice that experienced rMCAO/pMCAO via intraluminal suture insertions. A potential complication of the intraluminal suture insertion is iatrogenic subarachnoid hemorrhage (Schmid-Elsaesser et al., 1998), which may induce acute seizures secondary to brain ischemia. We thus conducted MCAO via electrocoagulation (pMCAOe) to address this potential complication. Of eight mice examined following pMCAOe, two mice exhibited NCS around 11 or 25 min following electrocoagulation surgery, but no animal showed CS within 24 h post surgery. These two mice were monitored unilaterally from ipsilateral hippocampal-cortical areas or bilaterally from ipsilateral hippocampal and contralateral cortical areas. In both cases, hippocampal discharges were remarkably longer and earlier in onset (by 10–13 s relative to cortical discharges (Figures 3A,B).

Collectively, of the total 37 NCS observed following rMCAO/pMCAO via intraluminal suture insertions, five events presented hippocampal discharge without or with weak cortical involvement but no event showed cortical discharge dominance over hippocampal discharges. In addition, RMS of ipsilateral or contralateral hippocampal discharges was always greater than that of cortical discharges. Moreover, in NCS observed following pMCAOe, hippocampal discharges were clearly advanced in onset relative to cortical discharges. We thus suggest that the hippocampal discharges may be a major and/or leading component of acute NCS when compared to corresponding cortical discharges in C57 black mice following MCAO.

#### Hippocampal Injury Observed From Mice With Acute Seizures Following MCAO

We performed brain histological examinations in 13 mice with detected NCS and CS or NCS alone following MCAO (*n* = 7 or 6) and in nine sham control mice. These mice were euthanized 16–20 h post-MCAO or 4–5 weeks following sham surgery. Coronal brain sections (30 μm thick) were stained with cresyl violet for general morphological assessments.

Ipsilateral brain injuries were observed in all 13 ischemic mice but no brain injury was found in the control mice. Hippocampal cellular injuries were evident in sections encompassing dorsal-middle hippocampal tissues and were more pronounced in CA1-CA3 areas relative to the dentate gyrus. These injuries featured diminished cytoplasmic staining, dark stained nuclei only and/or cell loss (Supplementary Figures S1A–C). CA1 cell densities of dorsal hippocampal areas were diminished in ipsilateral sections relative to contralateral sections or control sections (24.3 ± 0.9/100 pixels vs. 30.5 ± 1.0, 29.1 ± 1.1 or 30.4 ± 0.8/100 pixels; *p* < 0.05, ANOVA; Supplementary Figure S1E). In addition, area ratios of the ipsilateral vs. contralateral hemisphere were greater for ischemic mice than for control mice (108.8 ± 1.87% vs. 101.8 ± 0.6%; *p* = 0.005; Supplementary Figure S1D), implicating ipsilateral brain edema in the former. Injuries of ipsilateral lateral cortical and striatal areas, recognized by weak tissue staining and/or regional cell loss, were recognized in 6/13 ischemic mice. Overall, these observations are in keeping with previous studies (Furuya et al., 2004; Yuan et al., 2012; Nishijima et al., 2015) and further demonstrate hippocampal injury in C57 black mice following MCAO.

### Hippocampal Hyperexcitability Responses Observed Following HH *ex vivo*

We introduced HH episodes in mouse brain slices and examined resultant regional hyperexcitability responses. In these experiments, individual slices were first perfused with standard ACSF with 10 mM glucose and aeration by 95% O_2_. After baseline monitoring, slices were exposed to a modified ACSF with 1 mM glucose and aeration by 10% O_2_ for 12–13 min. To monitor hippocampal population activities, dual extracellular recordings were performed from CA3 and CA1 areas or CA3 and dentate gyrus areas, and synaptic field potentials were evoked by stimulating the CA3 stratum radiatum or oriens. To monitor cortical population activities, dual extracellular recordings were made from superficial and deep layers and synaptic field potentials were elicited by stimulating middle layers. Entorhinal population responses were recorded from the deep layer and afferent stimulation was applied locally. Whole-cell patch recordings were made from CA3 pyramidal neurons to examine their ionic and synaptic activities.

### Local Field Potentials Observed From Different Brain Regions Following HH

#### Observations From Brain Slices of Adult Mice

**Figure 4:**
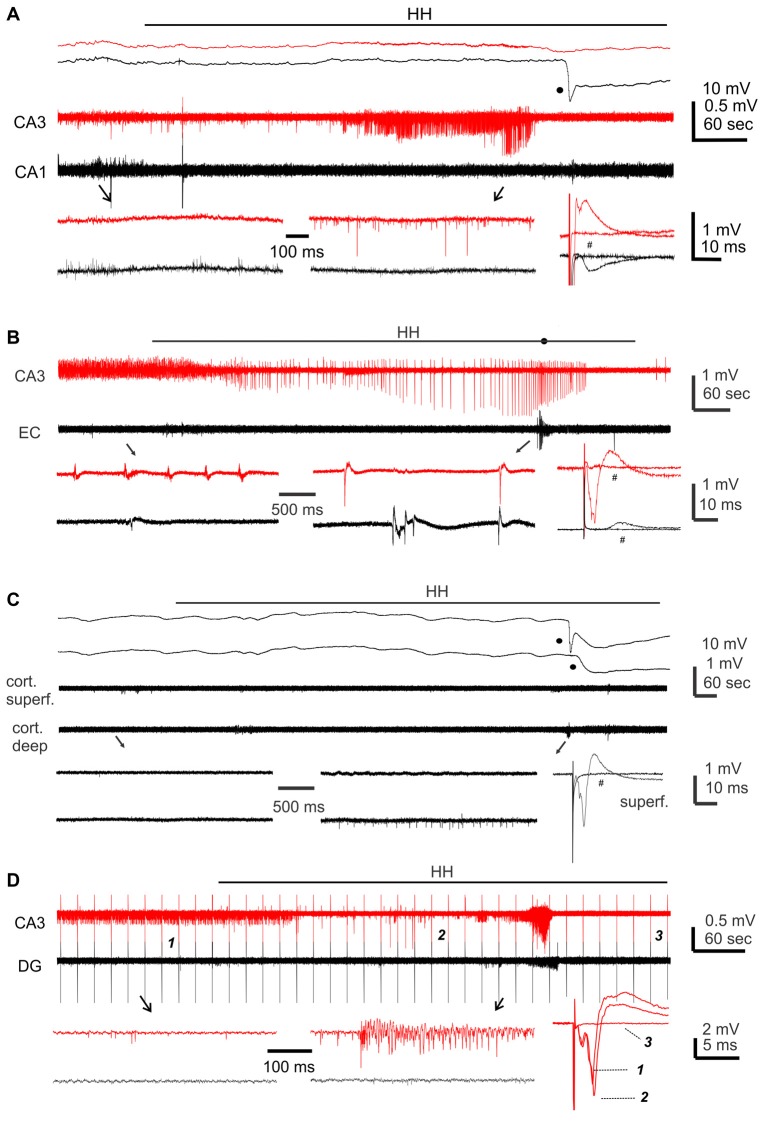
Regional field potentials observed from brain slices following hypoxic-hypoglycemic (HH). Dual extracellular recordings made from indicated areas in brain slices of adult **(A)** or young mice **(B–D)**. HH timings indicated by horizontal lines; anoxic depolarization (AD) responses denoted by filled circles. Field potentials evoked before and after HH termination (#) were superimposed at far right; illustrated traces were averages from 4 to 5 consecutive responses. **(A,C)** Top panel represent original signals that were sampled in a frequency range of 0–1,000 Hz; middle panel represent original signals after treatments with a band-pass filter (1–500 Hz); bottom panel represent expansion of arrowed data segments. **(B,D)** Signals similarly filtered and illustrated as in the middle and bottom panels of **(A)**. In **(D)** constant afferent stimuli were applied every 20 s continuously; arrowed segments were expanded and numbered responses were superimposed at far right.

We first conducted HH challenges in brain slices of adult mice (ages 5–9 months) and examined hippocampal and cortical responses. These were aimed to complement the ages and recording sites used in our *in vivo* experiments. A stimulating electrode was placed in the CA3 oriens area to elicit CA3 and CA1 field potentials or in the middle layer of the entorhinal or neocortical area to evoke local field potentials. Evoked synaptic field potentials were abolished in all CA3, CA1 and cortical areas when monitored 2–3 min after termination of HH (*n* = 20, 16 and seven slices from three to eight mice; Table 2; Figure 4A). No recovery in evoked responses was observed after re-perfusion with standard ACSF for up to 40 min, suggesting that the HH episode is a type of severe metabolic disturbance. SD, recognized as rapid negative (downward) shifts in extracellular potentials and thought to result from near-complete depolarization of neurons and glial cells through a complex of ionic mechanisms (Somjen, 2001; Ayata and Lauritzen, 2015), was observed following HH but not during baseline monitoring. The propensities of SD expression were similar in the CA3, CA1 and cortical areas, but SD latencies were shorter and SD magnitudes were smaller in the CA3 relative to the CA1 or cortical area (*p* < 0.05, Table 2).

**Table 2:** Regional changes observed in brain slices following hypoxic-hypoglycemic (HH) episodes.

Adult mice	Hippocampal CA3	Hippocampal CA1	Cerebral cortex
Evoked field potentials before HH (mV)	2.0 ± 0.2 (20)	0.3 ± 0.04 (16)	0.83 ± 0.12 (7)
Evoked field potentials following HH (mV)	abolished (20)	abolished (16)	abolished (7)
Slices exhibiting AD following HH	16 of 22	18 of 19	7 of 7
Latencies of AD (min)	282.4 ± 36.2 (16)*	362.8 ± 36.4 (18)	390.1 ± 24.8 (7)
Magnitudes of AD (mV)	4.1 ± 0.5 (16)*	7.0 ± 0.7 (18)	7.0 ± 0.5 (7)
**Young mice**	**Hippocampal CA3**	**Entorhinal cortex**	**Cerebral cortex**
Evoked field potentials before HH (mV)	1.6 ± 0.3 (15)	0.8 ± 0.2 (19)	0.7 ± 0.1 (13)
Evoked field potentials following HH (mV)	abolished (15)	abolished (19)	abolished (13)
Slices exhibiting AD following HH	1 of 34^#^	28 of 34	13 of 13
Latencies of AD (min)	167.0 (1)	394.9 ± 21.6 (28)	300.8 ± 21.1 (13)
Magnitudes of AD (mV)	3.4 (1)	7.2 ± 0.4 (28)	6.7 ± 0.6 (13)

Adult and young mice were 5–9 and 1–2 months of ages. HH episodes were induced by exposing slices to modified artificial cerebrospinal fluid (ACSF) with 1 mM glucose and 10% O_2_ aeration for 12–13 min. Synaptic field potentials were evoked by stimulating the CA3 oriens or the middle layer of entorhinal or neocortical areas. Latencies of anoxic depolarization (AD) were determined from the beginning of modified ACSF perfusion to onset of AD. AD magnitudes were determined by the onset-to-peak voltage of negative potential shifts. Data per each measure were collected from three to eight mice. Numbers in parentheses indicate slices examined. *CA3 vs. others, p < 0.05, One way ANOVA; ^#^CA3 vs. CA1 or cortex, p < 0.05, Fisher exact test.

**Figure 5:**
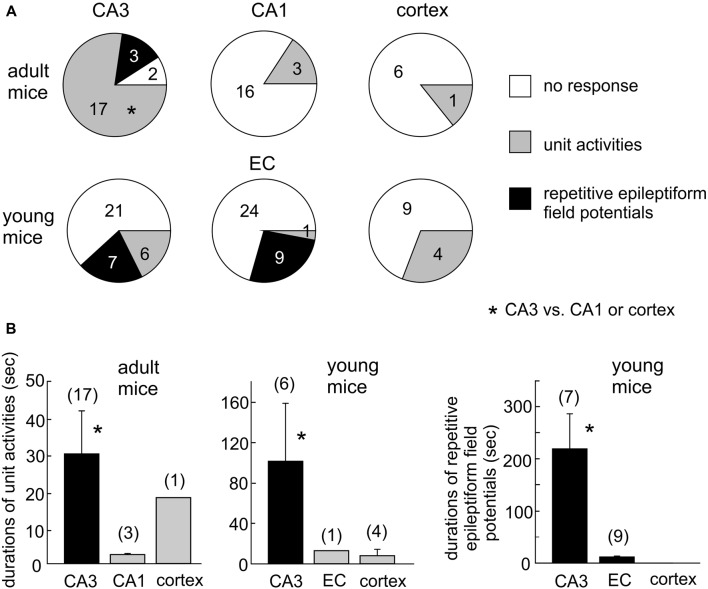
Summary of HH-induced hyperexcitable responses in CA3 and other regions. **(A)** Proportions of slices with or without regional unit activities or epileptiform potentials following HH. *CA3 vs. CA1 or cortex. **(B)** Durations of regional unit activities or epileptiform potentials. *CA3 vs. CA1, entorhinal cortical (EC) or cortical area. Data in each measures were collected from three to eight mice.

Single/multi-unit activities, recognized as clusters of extracellularly recorded spikes, were frequently observed following HH (Figure 4A). The propensity for exhibiting these unit activities was greater in the CA3 relative to the CA1 or cortical region, and the durations of CA3 unit activities were also longer (Figures 5A,B). Spontaneously occurring rhythmic field potentials, termed as *ex vivo* sharp waves (SPWs; Moradi-Chameh et al., 2014), were observed from the CA3 area in three slices. Baseline SPWs were 0.2–0.3 mV in amplitude and 0.4–2 Hz in frequency while faster rhythmic events lasting 49–410 s were observed following HH. Suppression of evoked field potentials, SD responses and unit activities were also observed following HH in the entorhinal area from three slices.

#### Observations From Brain Slices of Young Mice

We next conducted HH episodes in brain slices of young mice (ages 1–2 months) in an attempt to better preserve local brain circuitry and improve the probability of observing hyperexcitable local population responses (Bernard, 2006). Recordings and stimulating protocols were similar to those described above except that dual extracellular recordings were made from CA3 and entorhinal cortical (EC) areas in some experiments. These brain slices were also susceptible to the HH challenge as evoked synaptic field potentials were abolished in all CA3, entorhinal and cortical areas when monitored 2–3 min after termination of HH (15, 19 and 13 slices from four to seven mice; Table 2; Figures 4B–D). However, SD responses were rare in the CA3 compared to the entorhinal or cortical area (1/34 slices vs. 28/34 or 13/13 slices, *p* < 0.001, Fisher exact test; Table 2).

Single/multi-unit activities were also observed following HH (Figures 4B–D). The propensities for exhibiting these unit activities were similar among the CA3, entorhinal and neocortical areas examined, but the durations of unit activities were longer for the CA3 area (Figures 5A,B). CA3 SPWs were observed during baseline monitoring (0.8 ± 0.2 Hz, *n* = 14 slices; Figure 4B). These SPWs decreased in a few minutes following HH and then progressed to repetitive epileptiform field potentials with durations of 219.0 ± 67.4 s (*n* = 7; Figure 5B). Epileptiform field potentials were also observed from the entorhinal area but with shorter durations (14.6 ± 3.2 s *n* = 9 slices) relative to the CA3 events (Figure 5B). No epileptiform field potential was observed from the cortical area examined (13 slices from four mice; Figure 4C).

To examine the influence of the dentate gyrus on CA3 unit activities/epileptiform potentials, we made dual extracellular recordings from the cell body layers of the CA3 and dentate gyrus areas before and following HH. In six slices in which CA3 recordings revealed unit activities or mixed unit activities and epileptic field potentials following HH (durations of 40.2 ± 7.6 s), concurrent dentate gyrus recordings revealed no “spontaneous” events in four slices and brief unit activities in other two slices. The latter events were 4.7 or 18 s in duration and onset was delayed by 5 or 65 s relative to corresponding CA3 events (Figure 4D). Overall these observations suggest a minimal role of the dentate gyrus in initiating CA3 hyperexcitability responses following HH.

To examine the impact of glutamatergic activity on CA3 hyperexcitability responses, we examined the effects of HH in the presence of kynurenic acid, a general blocker of ionotropic glutamate receptors. Introduction of 3 mM kynurenic acid to ACSF greatly suppressed or abolished CA3 evoked field potentials in 11 slices of three mice examined. No CA3 unit activity/epileptiform potential was observed in these 11 slices following HH (in the presence of kynurenic acid) for up to 20 min. These observations were significantly different from those observed following HH the absence of kynurenic acid (13/34 slices, Figure 5A; *p* = 0.048), indicating the glutamatergic activity critical in generating CA3 unit activities/epileptiform field potentials following HH.

Together, the above observations suggest that the CA3 areas is more susceptible than other areas examined in exhibiting unit activities/epileptic field potentials following HH and that these CA3 hyperexcitable events are dependent upon the glutamatergic activity. We therefore focused on the CA3 area in following experiments and examined CA3 ionic and synaptic activities in brain slices of young mice.

#### CA3 Evoked Field Potentials Observed During HH

To examine changes in CA3 evoked synaptic field potentials during HH, we applied constant afferent stimuli every 20 s and recorded spontaneous and evoked field potentials continuously before and after HH challenge. An example of such recordings is illustrated in Figure 4D. Twin stimuli with an inter-stimulation interval of 30 ms were delivered to the CA3 stratum radiatum or oriens area to evoke CA3 field EPSPs (fEPSPs) and to assess paired enhancements; twin stimuli with an inter-stimulation interval of 4 ms were applied to the CA3 stratum oriens area to elicit local population spikes and to assess paired suppression.

**Figure 6:**
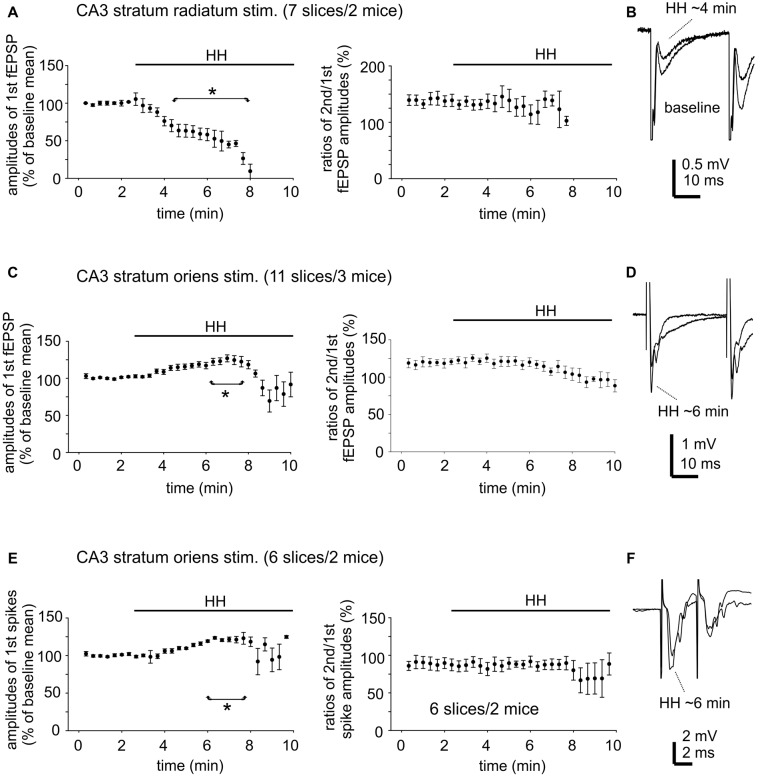
Changes in CA3 evoked field potentials observed during HH. **(A)** CA3 fEPSPs evoked every 20 s repetitively by paired stimulation (inter-stimulation interval of 30 ms) of the CA3 striatum radium area. The amplitudes of the 1st fEPSPs and paired enhancements (ratio of the 2nd vs. the 1st fEPSPs) were measured and normalized as percentiles of baseline means. HH timing indicated by horizontal lines. *, HH vs. baseline values. **(B)** Examples of evoked responses collected before and following HH for about 4 min. Averages from four consecutive responses were illustrated. **(C,D)** CA3 fEPSPs were evoked by similar paired stimulation of the CA3 striatum oriens area. Data were similarly analyzed and presented as in **(A,B)**. **(E,F)** CA3 population spikes evoked every 20 s repetitively by paired stimulation (inter-stimulation interval of 4 ms) of the CA3 striatum oriens area. Data were similarly analyzed and presented as in **(A,B)**.

CA3 fEPSPs evoked by stimulating the CA3 stratum radiatum were consistently suppressed following HH (seven slices from two mice). The amplitudes of the first fEPSPs were 1.1 ± 0.01 mV to 2.8 ± 0.01 mV during baseline monitoring, decreased to approximately 50% of baseline level following HH for 5-min and until they were abolished in following 2–3 min of HH (Figure 6A). CA3 fEPSPs evoked by stimulating the CA3 stratum oriens were initially enhanced and then suppressed following HH (11 slices from three mice). The amplitudes of the first fEPSPs were 1.0 ± 0.02 mV to 2.5 ± 0.02 mV during baseline monitoring, increased to 118%–122% above baseline level following HH for 5–6 min and then decreased (Figure 6C). Paired fEPSP enhancements in both groups of slices were not significantly altered following HH as compared with baseline measures (Figures 6B,D).

CA3 population spikes evoked by stimulating the CA3 stratum oriens were also initially enhanced and then suppressed following HH. The amplitudes of the first spikes were 2.3 ± 0.03 mV to 6.0 ± 0.06 mV during baseline monitoring (seven slices from two mice), increased to 120%–123% above baseline level following HH for about 6 min and then decreased (Figure 6E). Paired spike suppressions were similar between baseline and post-HH measures (Figure 6F).

Collectively, the above experiments showed variable effects of HH on CA3 evoked field potentials. As the CA3 unit activities/epileptiform field potentials occurred independently of applied afferent stimulation in a majority of the above experiments, we next examined “spontaneous” synaptic activities from individual CA3 pyramidal neurons as an alternative means of assessing CA3 circuitry excitability.

#### Changes in EPSCs/IPSCs Observed From CA3 Pyramidal Neurons During HH

**Figure 7:**
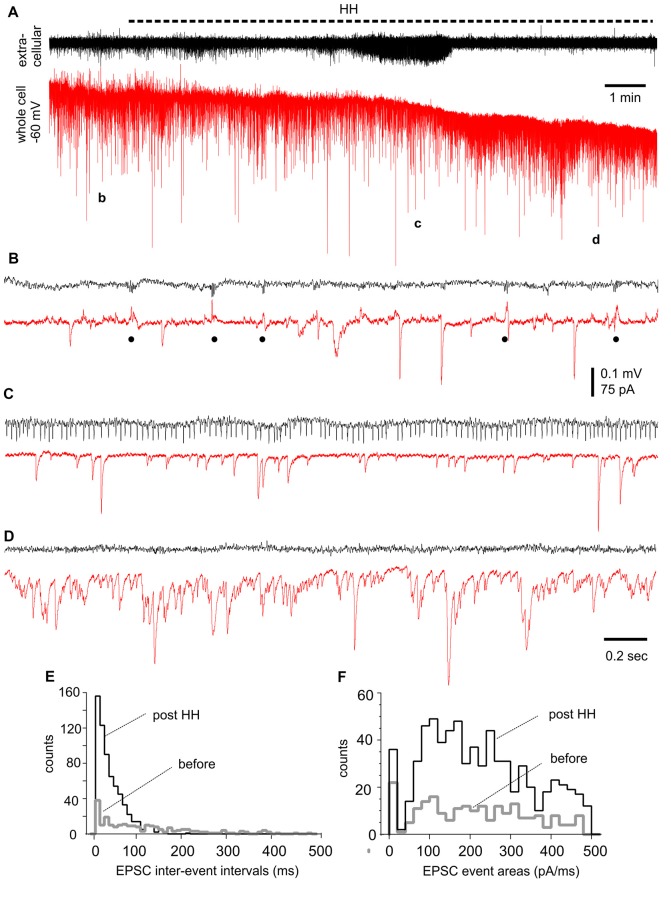
Synaptic currents observed from a CA3 pyramidal neuron before and following HH. **(A)** Traces collected via whole-cell patch recordings from a CA3 pyramidal neuron concurrent with extracellular recordings of local field potentials. The neuron was voltage-clamped at −60 mV. HH timing was indicated by a dashed line above traces. Data segments denoted by “b,” “c” and “d” were expanded in **(B–D)**, respectively. Filled circles in **(B)** indicate IPSCs. **(E,F)** Overlapped histograms show interval **(E)** and area **(F)** distributions of baseline and post-HH EPSCs (*n* = 149 and 554 events; bin size 10 ms or 20 pA/ms).

We performed voltage-clamp recordings to monitor synaptic currents in individual CA3 pyramidal neurons before and following HH. One example of such experiments is presented in Figure 7, where a CA3 pyramidal neuron was held at −60 mV during concomitant local extracellular recording. During baseline monitoring in standard ACSF, the neuron exhibited repetitive inward EPSCs with mono-phasic waveforms and variable amplitudes. Intermittent outward IPSCs with complex waveforms and in concurrence with extracellular SPWs were also noticeable (Figure 7B). Following HH for approximately 5 min, EPSCs appeared to be more frequent while IPSCs were diminished; a finding that complemented the appearance of unit activity in extracellular recordings (Figure 7C). EPSCs greatly increased in incidence and dominated with overlapped waveforms at about 10 min of HH (Figure 7D). EPSC inter-event intervals and charges (presented as EPSC areas) were measured from 30-s data segments collected during baseline monitoring and after approximately 10 min of HH exposure. These analyses showed great increases in EPSC frequency (as indicated by shortened intervals) and total charges (or EPSC areas) but similar distributions of mean EPSC charges per binned events (every 20 pA/ms; Figures 7E,F).

**Figure 8:**
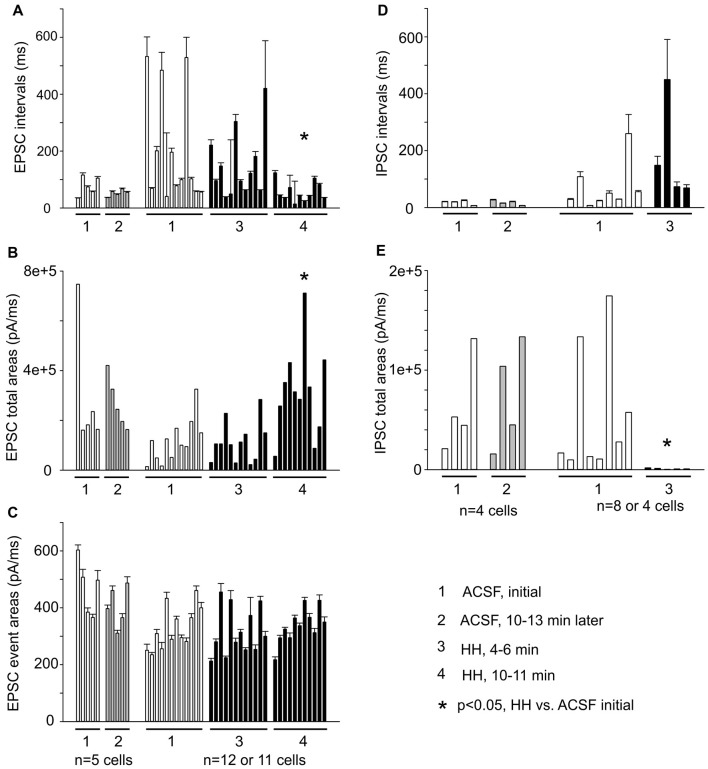
Summary of EPSC and IPSC measures from a group of CA3 pyramidal neurons (*n* = 5–12). **(A–C)** Measures of EPSC inter-event intervals, total charges and mean charges. **(D,E)** Measures of IPSC inter-event intervals and total charges. EPSC/IPSC total charges are presented as medians for individual neurons. Each column represents measures from 56 to 1,672 events/per neuron. *, post HH vs. baseline measures.

EPSC inter-event intervals and charges were similarly measured in 12 CA3 pyramidal neurons, showing a consistent decrease in EPSC intervals and an increase in EPSC total charges following HH but not substantial changes in the distributions of means EPSC charges/per binned events (Figures 8A–C). Measured at 4–5 min following HH, IPSC intervals were not significantly different from baseline measures, but IPSC total charges were reduced (Figures 8D,E). No IPSCs were detected following HH exposure for ≥5–6 min. In contrast to these changes, EPSCs and IPSCs were not significantly altered while being monitored in standard ACSF for 12–13 min (*n* = 4–5 CA3 neurons; Figures 8A–E). Overall, these experiments suggest excessive “spontaneous” EPSCs as a main outcome of CA3 pyramidal neurons in response to HH episodes.

#### Membrane Potentials/Currents and Spike Activities Observed From CA3 Pyramidal Neurons During HH

**Figure 9:**
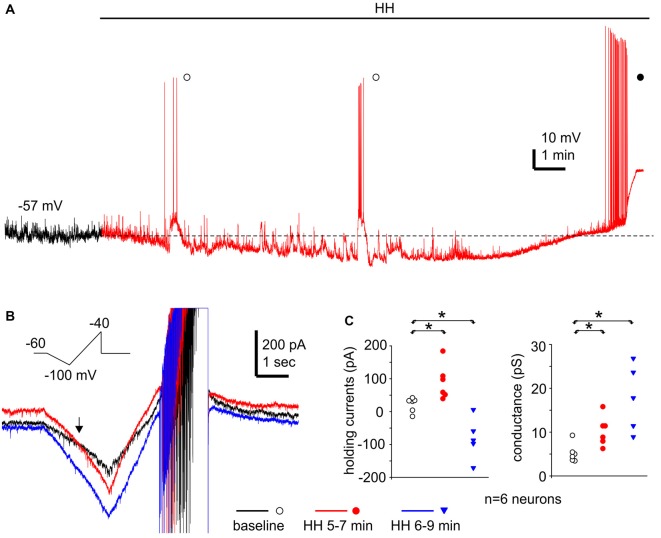
Membrane potentials/currents and spike activities observed from CA3 pyramidal neurons following HH. **(A)** A continuous voltage trace collected from a CA3 pyramidal neuron before (black) and following HH (red). HH timing indicated by a solid line above the trace; initial resting potential denoted by a dotted line; intermittent spikes superimposed onto EPSPs and repetitive spikes marked by open and filled circles respectively. **(B)** Ramp currents collected from another CA3 pyramidal neuron. This neuron was voltage-clamped at −60 mV and experienced repetitive voltage ramps (insert) every 20 s. Illustrated traces were averaged from four consecutive responses. **(C)** Holding currents and chord conductance measured from six CA3 pyramidal neurons of three mice. These neurons were clamped at −60 mV and experienced repetitive voltage ramps as illustrated in **(B)**. Measurements were made from averages from three consecutive responses. *, *p* < 0.05, HH vs. baseline.

Monitored via current-clamp recordings under baseline conditions, CA3 neurons displayed resting membrane potentials of −61.7 ± 1.0 mV, input resistance of 198 ± 12.5 MΩ, spike peak amplitudes of 95.1 ± 1.5 mV, spike half width of 1.0 ± 0.01 ms and spike voltage thresholds of −38.9 ± 1.2 mV (*n* = 16 neurons). These measurements were in keeping with previous studies of our lab on mouse CA3 pyramidal neurons (Wu et al., 2005a; Moradi-Chameh et al., 2014). In response to HH, CA3 pyramidal neurons showed hyperpolarizing and subsequent depolarizing shifts in membrane potentials, from −61.8 ± 1.9 mV to −71.0 ± 2.0 mV and then to −54.5 ± 2.9 mV following HH for 6–9 and 8–11 min respectively (*n* = 4 neurons, *p* = 0.015 and *p* = 0.081, HH vs. baseline; Figure 9A). Intermittent spikes superimposed onto large EPSPs and repetitive spikes at higher rates were observed during the hyperpolarization or depolarizing phases respectively (open and filled circles; Figure 9A). The latter spikes were 90.7 ± 3.9 mV in peak amplitudes and 1.14 ± 0.05 ms in half-width (52 spikes measured from three neurons), which were similar to the measures made from CA3 pyramidal neurons under baseline conditions.

We conducted voltage-clamp recordings and used a voltage ramp protocol (from −60 mV to −100 mV and then to −40 mV, 20 mV/sec, every 20 s; Chung et al., 1998) to study the ion currents underlying the membrane potential changes described above. When monitored at −60 mV and in response to the repetitive voltage ramps, CA3 pyramidal neurons displayed outward and then inward shifts in holding currents and increased conductance following HH (*n* = 6 neurons from three mice). Holding currents were −16 pA to 39 pA during baseline monitoring, and 37 pA to 181 pA and 3 pA to −174 pA following HH for 5–7 and 6–9 min respectively (*p* = 0.005 and 0.014; HH vs. baseline; Figures 9B,C). Chord conductance, measured from the voltage range of −60 mV to −100 mV (see “Materials and Methods” section), were in a range of 3.4–9.1 pS during baseline monitoring, 6.1–15.0 pS and 8.7–26.6 pS corresponding to the outward and inward current shits respectively (*p* = 0.011 and 0.004, HH vs. baseline; Figures 9B,C). The reversal potentials of the outward holding currents, estimated by the voltage at which baseline and post-HH ramp currents crossed over (Figure 9B; see “Materials and Methods” section), were −81.2 ± 2.3 mV. Spike currents induced positive ramp voltages (from −100 mV to −40 mV; Figure 9B) were consistently observed in all six neurons following HH for up to 10 min (red and blue traces; Figure 9B). These observations, together with the above current-clamp observations of “spontaneous” spikes following HH, suggest that CA3 pyramidal neurons were able to discharge during HH and that these discharges might contribute to the CA3 unit activities detected by extracellular recordings.

## Discussion

We explored the contributions of the hippocampal CA3 circuitry to post-ischemic seizures and hyperexcitable responses in mouse models *in vivo* and *ex vivo*. Three main observations emerged from the present experiments. First, acute NCS following MCAO manifested with robust hippocampal discharges. Second, hyperexcitable responses following HH were more prominent in the CA3 area than other hippocampal and cortical areas examined. Third, CA3 pyramidal neurons exhibited excessive EPSCs, attenuated IPSCs and spike activity in response to HH episodes.

### Hippocampal Contribution to Acute NCS in C57 Mice Following MCAO

In the rat model of MCAO, NCS are detectable within 3 h post-surgery and in 80%–90% of animals examined. EEG discharges recorded via epidural electrodes predominantly originated from the hemisphere ipsilateral to MCAO (Hartings et al., 2003; Williams et al., 2004, 2006; Karhunen et al., 2006; Lu et al., 2009, 2013; Cuomo et al., 2013; Kelly, 2017). In the present experiments, NCS were observed within 90 min post-surgery, in 25 of 67 (37%) mice examined, and featured hippocampal-cortical EEG discharges. Our observations and the previous studies are largely comparable with respect to NCS latencies, behavioral correlates and associated discharges, indicating NCS a common feature in rodent models of MCAO. However, NCS incidences are lower in our mouse model compared to the rat model (37% vs. 80%–90%). Reasons behind this discrepancy remain to be determined but species-related ischemia/seizure severities may be influencing factors. In particular, CS were observed in our mouse model but not documented in the rat model (see references above). We found that NCS always occurred prior to CS in mice that exhibited both types of seizures. In addition, suppression of ipsilateral hippocampal EEG was severer in mice with CS relative to those with NCS as the first seizure. Thus, a high propensity of CS and associated EEG suppression may partly explain a relatively low incidence of NCS in our model.

A consistent observation in our experiments was that hippocampal discharges were more robust than corresponding cortical discharges in all NCS events detected. In addition, NCS with hippocampal discharges alone or with minimal cortical discharges were observed in some cases, but no NCS events were observed with cortical discharges alone or with minimal hippocampal involvement. Moreover, hippocampal discharges were remarkably longer and advanced in onset relative to corresponding cortical discharges following pMCAOe. Overall, these observations support the notion that MCAO in mouse models may affect brain structures including the hippocampus beyond the territory of the MCA (Furuya et al., 2004; Yuan et al., 2012; Lin et al., 2013; Park et al., 2014; Nishijima et al., 2015). NCS with robust hippocampal discharges observed in a subset of mice might reflect differential disturbance of hippocampal blood flow by MCAO protocols in these animals relative to animals with CS alone. Inherited deficiency of posterior communicating artery, often observed in the C57 black mice and thought to an influencing factor of ischemic brain injury (Fujii et al., 1997; Kitagawa et al., 1998; Ozdemir et al., 1999; McColl et al., 2004; Adhami et al., 2006; El-Hayek et al., 2011), might also contribute to the robust hippocampal discharges observed in our model. Our histological observations of ipsilateral hippocampal injures in mice with NCS/CS are in line with the above view. However, these histological data are limited to reveal a causal relationship between NCS and brain injury as previously demonstrated in rats following MCAO (Williams et al., 2004, 2006; Lu et al., 2009, 2013; Cuomo et al., 2013). While our present experiments showed robust hippocampal discharges relative to cortical discharges, the issues remain as to whether other brain structures are involved in NCS genesis and if so, how these regional discharges are temporally related.

Despite the above limitations, our observations from a mouse model may be of clinical relevance. EEG monitoring is of great help in diagnosis of post-stroke seizures and in particular, NCS (Silverman et al., 2002; Jordan, 2004; Claassen et al., 2007; Mecarelli et al., 2011). Our present observations are in-line with clinical findings and further advocate routine EEG examinations for patients with stroke. In addition, our data suggest hippocampal contribution to post-ischemic NCS. As clinical skull EEG may not reliably detect discharges from hippocampal or other subcortical structures, the possibility of underdiagnosed post-stroke NCS may be considered in clinical EEG examinations. Furthermore, our present observations, together with previous experimental studies (Hartings et al., 2003; Williams et al., 2004, 2006; Karhunen et al., 2006, 2007; Lu et al., 2009, 2013; Cuomo et al., 2013; Paz et al., 2013; Tsenov et al., 2015; Lippmann et al., 2017; see review by Kelly, 2017), support the hypothesis that early detection of NCS and timely anticonvulsive treatments may suppress recurrent seizures and improve overall outcomes in patents with stroke.

### HH-Induced CA3 Hyperexcitability Responses

In slices obtained from both adult and young mice, the hippocampal CA3 region was prone to exhibiting hyperexcitable responses following HH compared to other regions examined. In particular, CA3 unit activities/epileptiform field potentials occurred prior to or the absence of corresponding events in the dentate gyrus, suggesting the former originated predominantly from the CA3 local circuitry. Our present observations are in line with a recent study demonstrating the mouse CA3 circuitry in generation of seizure-like events following hypoglycemic episodes *ex vivo* (Florez et al., 2015). Together these data indicate a high prevalence of CA3 hyperexcitability under conditions of metabolic disturbances (Carlen and Zhang, 2017).

The expression of CA3 hyperexcitable responses following HH was dependent upon the glutamatergic activity because HH in presence of kynurenic acid failed to induce CA3 unit activity/epileptiform field potential. However, changes in CA3 evoked field potentials were variable during HH, as CA3 fEPSPs evoked by stimulating the CA3 stratum radiatum were primarily suppressed whereas CA3 fEPSPs and population spikes evoked by stimulating the CA3 stratum oriens showed enhancements in varying degrees. The latter may implicate a subtle increase of glutamatergic transmission in the basal dendritic layer of CA3 pyramidal neurons, but the afferent stimulations used in our present experiments were limited in isolating CA3 sub-regional responses. As the overall effects of HH on CA3 evoked field potentials were inconclusive, we paid attention to repetitive EPSCs that were sampled from individual CA3 pyramidal neurons in the absence of applied afferent stimulations.

In all CA3 pyramidal neurons reliably monitored following HH, repetitive EPSCs were greatly increased in frequency and total charge as indicated by shortened inter-EPSC intervals and enlarged EPSC areas. Such EPSC increases were accompanied by attenuated or abolished IPSCs. These changes were not due to nonspecific effects of prolonged whole cell dialysis because repetitive EPSCs/IPSCs were not substantially altered in CA3 pyramidal neurons that were similarly monitored in standard ACSF. Overall these cellular observations suggest an increase of “spontaneous” glutamatergic activity in the CA3 circuitry following HH. However, the underlying mechanisms, particularly regarding the contributions of CA3 recurrent and the mossy fiber synapses (Witter, 2007; Le Duigou et al., 2014) as well as associated pre and/or postsynaptic alterations, remain to be investigated.

Previous studies may offer mechanistic insights relevant to our present observations. In particular, Ye et al. (2010) conducted briefer HH episodes in rat brain slices (ACSF without glucose and oxygenation for about 2 min) and examined resultant changes in repetitive EPSCs of CA1 pyramidal neurons. CA1 EPSCs were found to be greatly increased in amplitude and frequency following HH. Such EPSC increases were largely due to action potential-dependent “spontaneous” glutamate release from the Schaffer collateral pathway because HH in the presence of sodium channel blocker tetrodotoxin or HH applied to the isolated CA1 area caused only marginal increases in CA1 EPSCs. Our present observations are supportive of this study and further demonstrate that EPSCs of CA3 pyramidal neurons were increased following HH. Together these data suggest that enhanced “spontaneous” EPSCs may serve as a cellular readout of HH-induced hyperexcitability in the CA3 circuitry. Jalini et al. (2016) conducted similar HH episodes in mouse brain slices and examined resultant changes in presynaptic Ca^2+^ signals and evoked fEPSPs in the CA1 region. CA1 presynaptic Ca^2+^ signals were found to be elevated while CA1 evoked fEPSPs were suppressed following HH. Buffering of intracellular Ca^2+^ by membrane-permeant Ca^2+^ chelators lessened fEPSP suppression, suggesting that the former might negatively regulate CA1 evoked synaptic responses. Changes of CA3 presynaptic Ca^2+^ signals following HH remain to be examined in our model, but an early study reports elevations of intracellular Ca^2+^ following oxygen or glucose deprivation in the CA3 area of guinea pig hippocampal slices (Takata and Okada, 1995). Based on these studies, we speculate that potential elevations of CA3 presynaptic Ca^2+^ signals may be partly explain the variable changes in CA3 evoked fEPSPs and perhaps also the increase of “spontaneous” EPSCs we observed following HH.

Multiple factors may underlie the attenuated outward IPSCs we observed from CA3 pyramidal neurons following HH, including diminution of presynaptic GABA release and suppression of postsynaptic GABAa currents. Also, the intracellular Cl^−^ accumulation may have contributed to appearance of inward rather than outward IPSCs. To minimize influences by intracellular Cl^−^ accumulation and to examine GABAa IPSCs in relative isolation, we recorded CA3 pyramidal neurons using a “high [Cl^−^]” patch pipette solution (see “Materials and Methods” section) and in the presence of kynurenic acid (3 mM in ACSFs) before and following HH. Repetitive EPSCs were abolished and inward IPSCs and IPSCs superimposed with spike currents became frequent following application of kynurenic acid while CA3 pyramidal neurons were clamped at −60 mV before HH (*n* = 6 from two mice). Variable changes in inward IPSCs were observed following HH, with IPSC amplitudes and incidences being decreased in two neurons but unsubstantially unchanged in other four neurons. Considering such variability and that HH in the presence or absence of kynurenic acid may affect CA3 GABAergic transmission differently as judged by their effects on inducing CA3 hyperexcitable responses, we did not further examine inward, pharmacologically isolated IPCSs. While the underlying mechanisms are a topic of further investigation, the attenuated outward IPSCs observed from CA3 pyramidal neurons following HH are suggestive of weakened “spontaneous” GABAergic activity in the local circuitry.

### HH-Induced Changes in Membrane Potentials/Current and Spike Activities in CA3 Pyramidal Neurons

CA3 pyramidal neurons showed hyperpolarizing shifts in membrane potentials (current-clamp recordings) or outward shifts in holding currents (voltage-clamp recordings) following HH. Such hyperpolarizing or outward shifts were evident in the first several minutes of HH and then followed by depolarizing or inward shifts of varied degrees. The outward holding currents were reversed at about −80 mV and in accompany with increased chord conductance as assessed by the voltage ramp protocol. These measures, giving the ionic components of extra- and intracellular solutions used in our whole-cell experiments, are in agreement with a K^+^-dominated current. We therefore suggest that the initial ionic responses of CA3 pyramidal neurons to HH are dominated by a K^+^-mediated hyperpolarization. In view of hypoxic hyperpolarizing responses previously observed from CA3 pyramidal neurons of other rodent species (Hansen et al., 1982; Davis et al., 1986; Takata and Okada, 1995; Katchman and Hershkowitz, 1996; Wang et al., 2009), we speculate hypoxia is a major causal factor of the hyperpolarization observed in our model. However, further work is needed to determine whether elevated intracellular Ca^2+^, depleted intracellular ATP and/or activation of adenosine A1 receptors account for the HH-induced hyperpolarization and whether hypoglycemia episodes *per se* hyperpolarize CA3 pyramidal neurons in our model.

While their membrane potentials were hyperpolarized following HH, CA3 pyramidal neurons displayed intermittent spikes that occurred “spontaneously” and were superimposed onto large EPSPs. CA3 neurons exhibit repetitive spikes at relatively high frequencies while their membrane potentials were depolarized in the later phase of HH episodes. The peak amplitudes and half-widths of these spikes were similar to the measures made from CA3 pyramidal neurons under baseline conditions. Monitored via voltage clamp recordings and in response to positive voltage ramps, CA3 pyramidal neurons consistently exhibited spike currents during HH. Together these observations suggest that CA3 pyramidal neurons retain their ability to discharge during HH and that these discharges may be a significant component of the CA3 unit activities detected by extracellular recordings.

In summary, we provide convergent *in vivo* and *ex vivo* evidence suggesting a major contribution of the hippocampus, partially the CA3 circuitry, to acute seizures and hyperexcitable responses in mouse models of brain ischemia. While the mechanisms underlying regional initiation and spread of EEG discharges and EPSC/IPSC changes in CA3 neurons remain to be investigated, our experimental observations may be helpful for understanding acute post-stroke seizures seen in clinical practice.

## Author Contributions

HS, SMM, SMKM, JW and CW conducted experiments and data analysis. PC, JE, JF and LZ contributed to experimental design, data discussion and/or writing.

## Conflict of Interest Statement

The authors declare that the research was conducted in the absence of any commercial or financial relationships that could be construed as a potential conflict of interest.

## Supplementary Material

FIGURE S1 Hippocampal injury observed from mice following MCAO. **(A–C)** Images of brain sections collected from three mice at about 4 weeks following a sham surgery **(A)** or within 24 h post-rMCAO_90 min_ **(B)** or pMCAO **(C)**. Rectangle areas at left were enlarged in middle, square areas in middle are enlarged at right. Filled arrows denoted electrode tracks. **(D)** Bilateral hemispheric areas measured from individual sections and normalized as area ratios (%) of ipsilateral over contralateral hemispheres. Data collected from 3 to 4 adjacent sections per mouse and from 9 or 13 mice were pooled together and compared. **(E)** Cell body layers of hippocampal CA1 sectors (200 μm length) measured from individual sections and normalized as cell densities per 100 pixels. Data similarly analyzed as described for **(D)**. **(F)** Schematic illustrations correspond to coronal brain sections between 2.06 mm and 2.46 mm posterior to Bregma. Red squares indicate the correct localization of hippocampal electrode tracks in 11 post-MCAO mice examined. * and **, *p* < 0.05 or 0.001, post MCAO vs. control.
