# Supplementary figures and images for: Contributions of the Hippocampal CA3 Circuitry to Acute Seizures and Hyperexcitability Responses in Mouse Models of Brain Ischemia
Source: Front Cell Neurosci. 2018 Aug 29;12:278. doi: 10.3389/fncel.2018.00278 (PMC6123792; doi:10.3389/fncel.2018.00278)

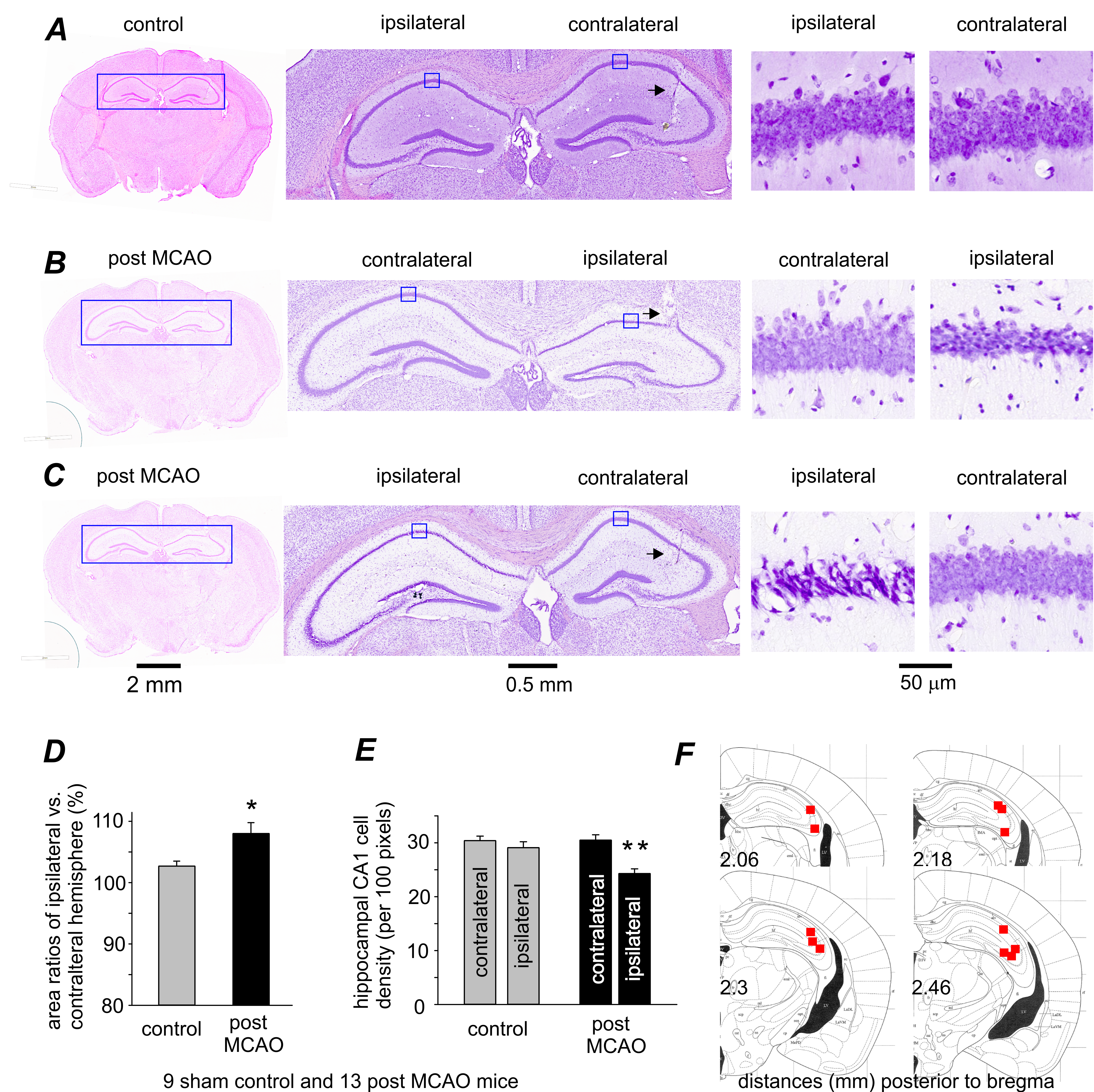

Supplement: FIGURE S1 — Hippocampal injury observed from mice following MCAO. (A–C) Images of brain sections collected from three mice at about 4 weeks following a sham surgery (A) or within 24 h post-rMCAO90 min (B) or pMCAO (C). Rectangle areas at left were enlarged in middle, square areas in middle are enlarged at right. Filled arrows denoted electrode tracks. (D) Bilateral hemispheric areas measured from individual sections and normalized as area ratios (%) of ipsilateral over contralateral hemispheres. Data collected from 3 to 4 adjacent sections per mouse and from 9 or 13 mice were pooled together and compared. (E) Cell body layers of hippocampal CA1 sectors (200 μm length) measured from individual sections and normalized as cell densities per 100 pixels. Data similarly analyzed as described for (D). (F) Schematic illustrations correspond to coronal brain sections between 2.06 mm and 2.46 mm posterior to Bregma. Red squares indicate the correct localization of hippocampal electrode tracks in 11 post-MCAO mice examined. * and **, p < 0.05 or 0.001, post MCAO vs. control. [file Image_1.TIF]
